# *N*-{2-[(4*S*)-4-*tert*-Butyl-4,5-dihydro-1,3-oxazol-2-yl]phen­yl}-5,6-diphenyl-1,2,4-triazin-3-amine

**DOI:** 10.1107/S1600536811005411

**Published:** 2011-02-19

**Authors:** Zbigniew Karczmarzyk, Ewa Wolińska, Andrzej Fruziński

**Affiliations:** a Department of Chemistry, University of Podlasie, ul. 3 Maja 54, 08-110 Siedlce, Poland; b Department of General and Ecological Chemistry, Technical University, ul. Żeromskiego 115, 90-924 Łódź, Poland

## Abstract

The title compound, C_28_H_27_N_5_O, was synthesized using palladium cross-coupling amination of 3-bromo-5,6-diphenyl-1,2,4-triazine with 2-[(4*S*)-4-*tert*-butyl-4,5-dihydro-1,3-oxazol-2-yl]aniline. The oxazoline ring is almost planar, with a maximum atomic deviation of 0.023 (5) Å. The phenyl rings make dihedral angles of 29.0 (1) and 54.6 (1)° with the triazine ring while the benzene ring makes a dihedral angle of 0.6 (1)° with the oxazoline ring. The conformation of the mol­ecule is influenced by strong intra­molecular N—H⋯N and weak C—H⋯N hydrogen bonds. In the crystal, screw-axis related mol­ecules are linked into supra­molecular chains by inter­molecular C—H⋯O hydrogen bonds. π–π stacking is observed between the oxazoline and triazine rings of adjacent mol­ecules, with a centroid–centroid distance of 3.749 (2) Å.

## Related literature

For applications of compounds containing a chiral oxazoline ring in asymmetric catalysis, see: Lindsey & Layton (2004 ▶); Desimoni *et al.* (2006 ▶); Hargaden & Guiry (2009 ▶). For related structures, see: Castro *et al.* (2001 ▶); Coeffard *et al.* (2009 ▶).
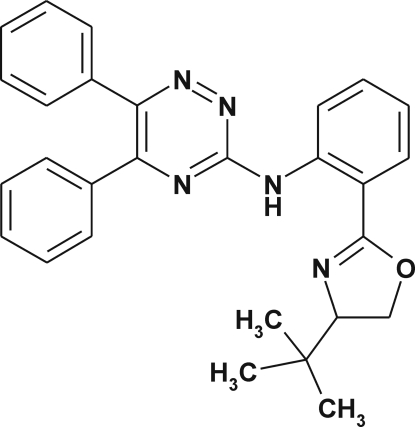


## Experimental

### 

#### Crystal data

C_28_H_27_N_5_O
*M* *_r_* = 449.55
Orthorhombic, 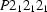

*a* = 6.3306 (2) Å
*b* = 16.9244 (6) Å
*c* = 22.5787 (8) Å
*V* = 2419.12 (14) Å^3^
*Z* = 4
Cu *K*α radiation
μ = 0.61 mm^−1^
*T* = 293 K
0.54 × 0.02 × 0.02 mm

#### Data collection

Bruker SMART APEXII CCD diffractometer
Absorption correction: multi-scan (*SADABS*; Bruker, 2005 ▶) *T*_min_ = 0.882, *T*_max_ = 1.000
28527 measured reflections
2625 independent reflections
1648 reflections with *I* > 2σ(*I*)
*R*_int_ = 0.090

#### Refinement

*R*[*F* ^2^ > 2σ(*F* ^2^)] = 0.050
*wR*(*F* ^2^) = 0.134
*S* = 1.05
2625 reflections
311 parameters
H atoms treated by a mixture of independent and constrained refinement
Δρ_max_ = 0.15 e Å^−3^
Δρ_min_ = −0.17 e Å^−3^

### 

Data collection: *APEX2* (Bruker, 2005 ▶); cell refinement: *SAINT* (Bruker, 2005 ▶); data reduction: *SAINT*; program(s) used to solve structure: *SHELXS97* (Sheldrick, 2008 ▶); program(s) used to refine structure: *SHELXL97* (Sheldrick, 2008 ▶); molecular graphics: *ORTEP-3 for Windows* (Farrugia, 1997 ▶); software used to prepare material for publication: *SHELXL97* and *WinGX* (Farrugia, 1999 ▶).

## Supplementary Material

Crystal structure: contains datablocks I, global. DOI: 10.1107/S1600536811005411/xu5158sup1.cif

Structure factors: contains datablocks I. DOI: 10.1107/S1600536811005411/xu5158Isup2.hkl

Additional supplementary materials:  crystallographic information; 3D view; checkCIF report

## Figures and Tables

**Table 1:** Hydrogen-bond geometry (Å, °)

*D*—H⋯*A*	*D*—H	H⋯*A*	*D*⋯*A*	*D*—H⋯*A*
N7—H7⋯N15	0.97 (4)	1.87 (5)	2.671 (4)	138 (4)
C13—H13⋯N2	0.93	2.31	2.919 (5)	122
C53—H53⋯O18^i^	0.93	2.54	3.250 (5)	133

Symmetry code: (i) 
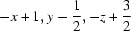
.

**Fig. 1.**
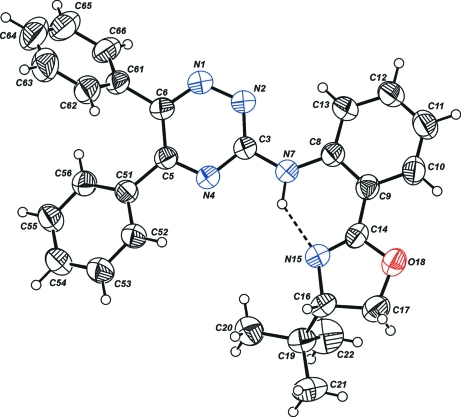
The molecular structure of (I), with atom labels and 50% probability displacement ellipsoids for non-H atoms.

**Fig. 2.**
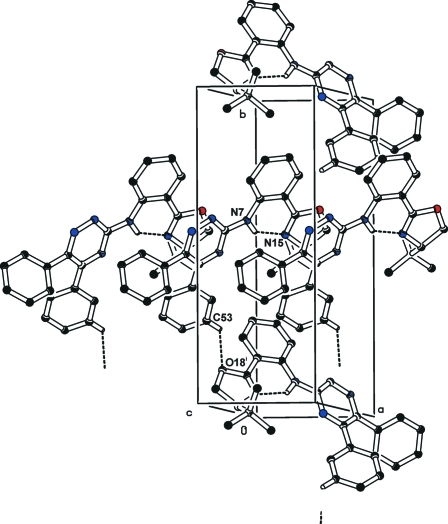
A view of the molecular packing in (I). Dashed lines indicate weak C—H···O intermolecular interaction [symmetry code: (i) –x+1, y–1/2, –z+3/2].

## supplementary crystallographic information

### Comment

Compounds containing a chiral oxazoline ring have proven to be one of the most successful ligand classes for asymmetric catalysis. A diverse range of di-, tri- and tetradentate oxazoline ligands incorporating various heteroatoms and specific structural features have been synthesized and used in a wide range of metal catalyzed asymmetric processes (Desimoni *et al.*, 2006; Hargaden *et al.*, 2009). Introduction of 1,2,4-triazine ring into ligand structure can significantly increase ligand binding properties, since 1,2,4-triazine is known as a good metal chelator (Lindsey *et al.*, 2004). Due to our interest in developing new oxazoline-based ligands the titled compound was synthesized and its application in asymmetric catalysis is currently under investigation.

The central secondary N7-amine group is planar with the sum of the angles around N atom of 359.1°. The unusually large C3—N7—C8 angle of 131.1 (3)° is constrained by the strong N7—H7···N15 intramolecular hydrogen bond (Table 1), which forced a *cis*-*cis* conformation of the amine spacer between 1,2,4-triazine ring and the (oxazolyl)phenyl group with the torsion angles N2—C3—N7—C8 and C3—N7—C8—C13 of 3.9 (6) and –12.4 (6)°, respectively. The similar geometry and conformation of the [(oxazolyl)phenyl]amine subunit have been reported in closely related structures (Castro *et al.*, 2001; Coeffard *et al.*, 2009). The 5- and 6-phenyl substituents of the 1,2,4-triazine ring are inclined to its mean plane with the dihedral angle of 29.0 (1) and 54.6 (1)°, respectively.

In the crystal structure, Fig. 2, the screw-related molecules are linked into chains along the [010] direction by C53—H53···O18 intermolecular hydrogen bond (Table 1). Additionally, the *π*-electron systems of the oxazoline and triazine rings belonging to the translation-related molecules overlap each other, with centroid-to-centroid separation of 3.749 (2) Å between the oxazoline ring at (*x*, *y*, *z*) and triazine ring at (1+*x*, *y*, *z*), and triazine ring at (*x*, *y*, *z*) and oxazoline ring at (–1+*x*, *y*, *z*). The *π*···*π* distances are 3.2389 (16) and 3.4927 (13) Å, respectively.

### Experimental

The titled compound was synthesized using palladium cross-coupling amination of 5,6-diphenyl-3-bromo-1,2,4-triazine with readily available 2-[(4*S*)-4-*tert*-butyl-4,5-dihydro-1,3-oxazol-2-yl]aniline as the key step. An oven dried three-necked flask was washed with argon and charged with Pd_2_dba_3_ (45.7 mg, 0.05 mmol), Xantphos (57.8 mg, 0.1 mmol), 2-[(4*S*)-4-*tert*-butyl-4,5-dihydro-1,3-oxazol-2-yl]aniline (0.131 g, 0.6 mmol), 3-bromo-5,6-diphenyl-1,2,4-triazine (155 mg, 0.5 mmol) and K_2_CO_3_ (1.38g, 10 mmol). Then, the flask was evacuated and backfilled with argon. Dioxane (10 ml) was added trough the septum. The mixture was refluxed for 24 hours. After cooling, the solid material was filtered off and washed with CH_2_Cl_2_. The solvent was evaporated, and the resulting crude product was purified by column chromatography using hexanes/ethyl acetate (10:1) as eluent. Product was recrystalized from ethanol to give 5,6-diphenyl-3-{2-[(4*S*)-4-*tert*-butyl-4,5-dihydro-1,3-oxazol-2-yl]phenyl}amino-1,2,4-triazine as a yellow crystals; yield: 0.095 g, 42%; mp 489-490 K; [*α*]_D_^20 ^of –15.69°. Crystals suitable for X-ray diffraction analysis were grown by slow evaporation of a methanol solution.

### Refinement

In the absence of significant anomalous scattering, Friedel pairs were merged and the absolute configuration was assigned from the absolute configuration of started 2-[(4*S*)-4-*tert*-butyl-4,5-dihydro-1,3-oxazol-2-yl]aniline. All H atom were located by difference Fourier synthesis. N-bound H atom was refined freely. The remaining H atoms were treated as riding on their C atoms, with C—H distances of 0.93 (aromatic) and 0.96 Å (CH_3_). All H atoms were assigned *U*_iso_(H) values of 1.5*U*_eq_(N,C).

### Crystal data

**Table:**

C_28_H_27_N_5_O	*D*_x_ = 1.234 Mg m^−^^3^
*M**_r_* = 449.55	Melting point = 489–490 K
Orthorhombic, *P*2_1_2_1_2_1_	Cu *K*α radiation, λ = 1.54178 Å
Hall symbol: P 2ac 2ab	Cell parameters from 1210 reflections
*a* = 6.3306 (2) Å	θ = 4.6–30.0°
*b* = 16.9244 (6) Å	µ = 0.61 mm^−^^1^
*c* = 22.5787 (8) Å	*T* = 293 K
*V* = 2419.12 (14) Å^3^	Needle, yellow
*Z* = 4	0.54 × 0.02 × 0.02 mm
*F*(000) = 952	

### Data collection

**Table:**

Bruker SMART APEXII CCD diffractometer	2625 independent reflections
Radiation source: fine-focus sealed tube	1648 reflections with *I* > 2σ(*I*)
graphite	*R*_int_ = 0.090
ω scans	θ_max_ = 70.2°, θ_min_ = 3.3°
Absorption correction: multi-scan (*SADABS*; Bruker, 2005)	*h* = −7→6
*T*_min_ = 0.882, *T*_max_ = 1.000	*k* = −20→20
28527 measured reflections	*l* = −27→27

### Refinement

**Table:**

Refinement on *F*^2^	Secondary atom site location: difference Fourier map
Least-squares matrix: full	Hydrogen site location: difference Fourier map
*R*[*F*^2^ > 2σ(*F*^2^)] = 0.050	H atoms treated by a mixture of independent and constrained refinement
*wR*(*F*^2^) = 0.134	*w* = 1/[σ^2^(*F*_o_^2^) + (0.0683*P*)^2^] where *P* = (*F*_o_^2^ + 2*F*_c_^2^)/3
*S* = 1.05	(Δ/σ)_max_ < 0.001
2625 reflections	Δρ_max_ = 0.15 e Å^−^^3^
311 parameters	Δρ_min_ = −0.17 e Å^−^^3^
0 restraints	Extinction correction: *SHELXL97* (Sheldrick, 2008), Fc^*^=kFc[1+0.001xFc^2^λ^3^/sin(2θ)]^-1/4^
Primary atom site location: structure-invariant direct methods	Extinction coefficient: 0.0053 (5)

### Special details

**Table:**

Experimental. ^1^H NMR (400 MHz, CDCl3) *δ*: 1.05 (*s*, 9H, (CH_3_)_3_C), 4.25–4.35 (*m*, 2H, (CH_2_O and CHN), 4.45–4.50 (*m*, 1H, CH_2_O), 7.11 (*t*, 1H, *J* = 7.6 Hz, Ph), 7.31–7.37 (*m*, 5H, Ph), 7.42–7.44 (*m*, 1H, Ph), 7.50–7.52 (*m*, 2H, Ph), 7.56–7.60 (*m*, 3H, Ph), 7.91 (*d*, 1H, *J* = 7.6 Hz, Ph), 8.89 (*d*, 1H, *J* = 8.4 Hz, Ph), 12.98 (*s*, 1H, NH); ^13^C NMR (50 MHz, CDCl_3_) *δ*: 25.9, 34.0, 67.5, 76.1, 112.8, 118.9, 120.7, 128.2, 128.3, 128.6, 129.2, 129.3, 129.8, 130.4, 132.3, 136.0, 136.1, 140.8, 150.8, 156.0, 158.8, 163.4; Analysis calculated for C_28_H_27_N_5_O: C 74.81; H 6.05; N 15.58; found: C 74.79; H 6.03; N 15.51.
Geometry. All esds (except the esd in the dihedral angle between two l.s. planes) are estimated using the full covariance matrix. The cell esds are taken into account individually in the estimation of esds in distances, angles and torsion angles; correlations between esds in cell parameters are only used when they are defined by crystal symmetry. An approximate (isotropic) treatment of cell esds is used for estimating esds involving l.s. planes.
Refinement. Refinement of F^2^ against ALL reflections. The weighted R-factor wR and goodness of fit S are based on F^2^, conventional R-factors R are based on F, with F set to zero for negative F^2^. The threshold expression of F^2^ > 2sigma(F^2^) is used only for calculating R-factors(gt) etc. and is not relevant to the choice of reflections for refinement. R-factors based on F^2^ are statistically about twice as large as those based on F, and R- factors based on ALL data will be even larger.

### Fractional atomic coordinates and isotropic or equivalent isotropic displacement parameters (Å^2^)

**Table:**

	*x*	*y*	*z*	*U*_iso_*/*U*_eq_	
O18	0.9330 (4)	0.61773 (16)	0.78505 (12)	0.0793 (8)	
N1	−0.0592 (5)	0.54476 (19)	0.97426 (12)	0.0735 (9)	
N2	0.0979 (5)	0.58502 (18)	0.94731 (14)	0.0728 (9)	
N4	0.1210 (5)	0.48257 (17)	0.87487 (12)	0.0640 (8)	
N7	0.3575 (5)	0.58275 (18)	0.87252 (13)	0.0697 (9)	
H7	0.411 (8)	0.548 (2)	0.8420 (18)	0.105*	
N15	0.6459 (5)	0.54077 (19)	0.79214 (13)	0.0716 (9)	
C3	0.1856 (6)	0.5505 (2)	0.90057 (16)	0.0611 (9)	
C5	−0.0365 (6)	0.4448 (2)	0.90072 (15)	0.0578 (9)	
C6	−0.1237 (6)	0.4751 (2)	0.95379 (15)	0.0613 (9)	
C8	0.4787 (6)	0.6496 (2)	0.88589 (15)	0.0647 (10)	
C9	0.6707 (6)	0.6585 (2)	0.85444 (16)	0.0637 (10)	
C10	0.7987 (7)	0.7239 (2)	0.86881 (17)	0.0784 (11)	
H10	0.9268	0.7306	0.8493	0.118*	
C11	0.7389 (8)	0.7781 (3)	0.91095 (19)	0.0894 (14)	
H11	0.8266	0.8205	0.9200	0.134*	
C12	0.5491 (8)	0.7693 (2)	0.93957 (18)	0.0828 (12)	
H12	0.5080	0.8064	0.9677	0.124*	
C13	0.4183 (8)	0.7061 (2)	0.92720 (16)	0.0780 (12)	
H13	0.2893	0.7014	0.9466	0.117*	
C14	0.7399 (6)	0.6022 (2)	0.80992 (16)	0.0647 (10)	
C16	0.7797 (6)	0.5003 (2)	0.74766 (16)	0.0668 (10)	
H16	0.8305	0.4509	0.7653	0.100*	
C17	0.9690 (7)	0.5565 (3)	0.74209 (19)	0.0855 (12)	
H171	1.0999	0.5289	0.7504	0.128*	
H172	0.9764	0.5786	0.7025	0.128*	
C19	0.6617 (6)	0.4794 (2)	0.69076 (17)	0.0741 (11)	
C20	0.4820 (8)	0.4227 (3)	0.7063 (2)	0.1036 (15)	
H201	0.3925	0.4466	0.7357	0.155*	
H202	0.5398	0.3744	0.7217	0.155*	
H203	0.4007	0.4115	0.6714	0.155*	
C21	0.8171 (9)	0.4373 (3)	0.64968 (19)	0.1075 (16)	
H211	0.8815	0.3940	0.6704	0.161*	
H212	0.9243	0.4737	0.6371	0.161*	
H213	0.7429	0.4176	0.6157	0.161*	
C22	0.5743 (10)	0.5535 (3)	0.6608 (2)	0.1179 (18)	
H221	0.6884	0.5888	0.6516	0.177*	
H222	0.4769	0.5793	0.6870	0.177*	
H223	0.5026	0.5389	0.6250	0.177*	
C51	−0.1113 (6)	0.3731 (2)	0.86873 (14)	0.0588 (9)	
C52	0.0351 (7)	0.3342 (2)	0.83248 (16)	0.0694 (11)	
H52	0.1747	0.3513	0.8317	0.104*	
C53	−0.0247 (7)	0.2709 (2)	0.7979 (2)	0.0833 (12)	
H53	0.0742	0.2456	0.7741	0.125*	
C54	−0.2320 (7)	0.2451 (2)	0.7986 (2)	0.0828 (12)	
H54	−0.2729	0.2026	0.7751	0.124*	
C55	−0.3780 (7)	0.2826 (2)	0.83435 (18)	0.0802 (12)	
H55	−0.5171	0.2650	0.8351	0.120*	
C56	−0.3186 (6)	0.3463 (2)	0.86901 (16)	0.0684 (10)	
H56	−0.4184	0.3713	0.8927	0.103*	
C61	−0.2858 (6)	0.4352 (2)	0.99015 (15)	0.0676 (10)	
C62	−0.2523 (8)	0.3591 (2)	1.01024 (18)	0.0882 (13)	
H62	−0.1272	0.3328	1.0013	0.132*	
C63	−0.4080 (12)	0.3219 (4)	1.0441 (2)	0.119 (2)	
H63	−0.3863	0.2707	1.0579	0.179*	
C64	−0.5935 (11)	0.3607 (5)	1.0572 (2)	0.133 (3)	
H64	−0.6972	0.3355	1.0794	0.200*	
C65	−0.6258 (9)	0.4369 (4)	1.0375 (2)	0.1133 (19)	
H65	−0.7513	0.4631	1.0460	0.170*	
C66	−0.4700 (6)	0.4739 (3)	1.00486 (17)	0.0838 (13)	
H66	−0.4897	0.5258	0.9926	0.126*	

### Atomic displacement parameters (Å^2^)

**Table:**

	*U*^11^	*U*^22^	*U*^33^	*U*^12^	*U*^13^	*U*^23^
O18	0.0671 (17)	0.0869 (18)	0.0839 (17)	−0.0114 (14)	0.0118 (14)	0.0045 (16)
N1	0.079 (2)	0.074 (2)	0.068 (2)	−0.0071 (18)	0.0151 (17)	−0.0099 (17)
N2	0.078 (2)	0.072 (2)	0.0678 (19)	−0.0101 (17)	0.0166 (17)	−0.0130 (17)
N4	0.0636 (19)	0.0691 (19)	0.0592 (17)	−0.0067 (16)	0.0089 (15)	−0.0032 (15)
N7	0.072 (2)	0.070 (2)	0.067 (2)	−0.0153 (17)	0.0156 (17)	−0.0113 (16)
N15	0.074 (2)	0.073 (2)	0.0673 (19)	−0.0073 (18)	0.0152 (17)	−0.0071 (17)
C3	0.062 (2)	0.063 (2)	0.058 (2)	−0.0057 (19)	0.0050 (18)	−0.0041 (19)
C5	0.059 (2)	0.060 (2)	0.054 (2)	0.0002 (18)	0.0035 (17)	0.0006 (17)
C6	0.066 (2)	0.067 (2)	0.051 (2)	−0.0009 (19)	0.0042 (18)	−0.0020 (18)
C8	0.072 (2)	0.064 (2)	0.058 (2)	−0.007 (2)	0.0023 (19)	0.0023 (19)
C9	0.066 (2)	0.065 (2)	0.059 (2)	−0.0057 (19)	−0.0018 (18)	0.0071 (19)
C10	0.080 (3)	0.078 (3)	0.076 (3)	−0.022 (2)	−0.002 (2)	0.009 (2)
C11	0.110 (4)	0.078 (3)	0.080 (3)	−0.026 (3)	−0.009 (3)	−0.005 (2)
C12	0.103 (3)	0.074 (3)	0.071 (3)	−0.013 (3)	0.001 (3)	−0.012 (2)
C13	0.093 (3)	0.075 (3)	0.066 (2)	−0.013 (2)	0.003 (2)	−0.014 (2)
C14	0.059 (2)	0.073 (2)	0.061 (2)	−0.007 (2)	0.0077 (19)	0.009 (2)
C16	0.066 (2)	0.074 (2)	0.061 (2)	0.0077 (19)	0.0127 (19)	0.0087 (19)
C17	0.075 (3)	0.101 (3)	0.080 (3)	−0.005 (3)	0.014 (2)	−0.003 (3)
C19	0.078 (3)	0.079 (3)	0.065 (2)	0.008 (2)	0.003 (2)	0.001 (2)
C20	0.095 (3)	0.108 (4)	0.108 (3)	−0.017 (3)	0.008 (3)	−0.024 (3)
C21	0.109 (4)	0.136 (4)	0.077 (3)	0.014 (3)	0.023 (3)	−0.016 (3)
C22	0.123 (4)	0.128 (4)	0.103 (4)	0.032 (4)	−0.023 (3)	0.025 (3)
C51	0.065 (2)	0.056 (2)	0.056 (2)	−0.0021 (18)	−0.0004 (18)	−0.0003 (17)
C52	0.071 (3)	0.063 (2)	0.075 (3)	0.002 (2)	0.006 (2)	−0.008 (2)
C53	0.086 (3)	0.070 (3)	0.094 (3)	0.003 (2)	0.010 (3)	−0.018 (2)
C54	0.088 (3)	0.072 (3)	0.088 (3)	−0.004 (2)	−0.008 (3)	−0.017 (2)
C55	0.076 (3)	0.073 (3)	0.092 (3)	−0.006 (2)	−0.008 (2)	−0.014 (2)
C56	0.064 (2)	0.073 (2)	0.068 (2)	−0.004 (2)	0.0008 (19)	−0.002 (2)
C61	0.072 (3)	0.080 (3)	0.051 (2)	−0.015 (2)	0.0057 (18)	0.000 (2)
C62	0.110 (4)	0.088 (3)	0.067 (2)	−0.022 (3)	0.003 (2)	0.008 (2)
C63	0.159 (6)	0.117 (4)	0.081 (3)	−0.054 (4)	−0.001 (4)	0.020 (3)
C64	0.126 (5)	0.199 (7)	0.075 (3)	−0.074 (6)	0.013 (4)	0.007 (4)
C65	0.086 (4)	0.180 (6)	0.074 (3)	−0.032 (4)	0.015 (3)	−0.001 (4)
C66	0.065 (3)	0.125 (4)	0.062 (2)	−0.011 (3)	0.009 (2)	−0.005 (2)

### Geometric parameters (Å, °)

**Table:**

O18—C14	1.370 (4)	C19—C21	1.529 (6)
O18—C17	1.437 (5)	C20—H201	0.9600
N1—C6	1.330 (4)	C20—H202	0.9600
N1—N2	1.351 (4)	C20—H203	0.9600
N2—C3	1.328 (4)	C21—H211	0.9600
N4—C5	1.320 (4)	C21—H212	0.9600
N4—C3	1.351 (4)	C21—H213	0.9600
N7—C3	1.372 (4)	C22—H221	0.9600
N7—C8	1.399 (4)	C22—H222	0.9600
N7—H7	0.97 (4)	C22—H223	0.9600
N15—C14	1.264 (5)	C51—C56	1.389 (5)
N15—C16	1.482 (5)	C51—C52	1.401 (5)
C5—C6	1.415 (5)	C52—C53	1.379 (5)
C5—C51	1.490 (5)	C52—H52	0.9300
C6—C61	1.478 (5)	C53—C54	1.383 (6)
C8—C13	1.390 (5)	C53—H53	0.9300
C8—C9	1.416 (5)	C54—C55	1.382 (6)
C9—C10	1.410 (5)	C54—H54	0.9300
C9—C14	1.452 (5)	C55—C56	1.383 (5)
C10—C11	1.375 (6)	C55—H55	0.9300
C10—H10	0.9300	C56—H56	0.9300
C11—C12	1.372 (6)	C61—C66	1.377 (5)
C11—H11	0.9300	C61—C62	1.382 (5)
C12—C13	1.381 (6)	C62—C63	1.397 (7)
C12—H12	0.9300	C62—H62	0.9300
C13—H13	0.9300	C63—C64	1.378 (9)
C16—C19	1.527 (5)	C63—H63	0.9300
C16—C17	1.536 (6)	C64—C65	1.380 (8)
C16—H16	0.9800	C64—H64	0.9300
C17—H171	0.9700	C65—C66	1.381 (6)
C17—H172	0.9700	C65—H65	0.9300
C19—C22	1.528 (5)	C66—H66	0.9300
C19—C20	1.530 (6)		
			
C14—O18—C17	106.3 (3)	C20—C19—C21	109.0 (3)
C6—N1—N2	121.1 (3)	C19—C20—H201	109.5
C3—N2—N1	116.3 (3)	C19—C20—H202	109.5
C5—N4—C3	116.8 (3)	H201—C20—H202	109.5
C3—N7—C8	131.1 (3)	C19—C20—H203	109.5
C3—N7—H7	111 (3)	H201—C20—H203	109.5
C8—N7—H7	117 (3)	H202—C20—H203	109.5
C14—N15—C16	109.1 (3)	C19—C21—H211	109.5
N2—C3—N4	126.1 (3)	C19—C21—H212	109.5
N2—C3—N7	121.5 (3)	H211—C21—H212	109.5
N4—C3—N7	112.4 (3)	C19—C21—H213	109.5
N4—C5—C6	119.6 (3)	H211—C21—H213	109.5
N4—C5—C51	114.8 (3)	H212—C21—H213	109.5
C6—C5—C51	125.6 (3)	C19—C22—H221	109.5
N1—C6—C5	119.7 (3)	C19—C22—H222	109.5
N1—C6—C61	115.2 (3)	H221—C22—H222	109.5
C5—C6—C61	125.1 (3)	C19—C22—H223	109.5
C13—C8—N7	123.4 (3)	H221—C22—H223	109.5
C13—C8—C9	119.9 (3)	H222—C22—H223	109.5
N7—C8—C9	116.7 (3)	C56—C51—C52	118.3 (3)
C10—C9—C8	117.5 (4)	C56—C51—C5	124.4 (3)
C10—C9—C14	120.0 (4)	C52—C51—C5	117.1 (3)
C8—C9—C14	122.4 (3)	C53—C52—C51	120.9 (4)
C11—C10—C9	121.7 (4)	C53—C52—H52	119.5
C11—C10—H10	119.2	C51—C52—H52	119.5
C9—C10—H10	119.2	C52—C53—C54	120.0 (4)
C10—C11—C12	119.6 (4)	C52—C53—H53	120.0
C10—C11—H11	120.2	C54—C53—H53	120.0
C12—C11—H11	120.2	C53—C54—C55	119.7 (4)
C11—C12—C13	121.0 (4)	C53—C54—H54	120.1
C11—C12—H12	119.5	C55—C54—H54	120.1
C13—C12—H12	119.5	C54—C55—C56	120.4 (4)
C12—C13—C8	120.3 (4)	C54—C55—H55	119.8
C12—C13—H13	119.9	C56—C55—H55	119.8
C8—C13—H13	119.9	C55—C56—C51	120.6 (4)
N15—C14—O18	116.6 (4)	C55—C56—H56	119.7
N15—C14—C9	128.1 (3)	C51—C56—H56	119.7
O18—C14—C9	115.3 (3)	C66—C61—C62	119.6 (4)
N15—C16—C19	113.4 (3)	C66—C61—C6	120.3 (4)
N15—C16—C17	102.4 (3)	C62—C61—C6	120.1 (4)
C19—C16—C17	117.1 (3)	C61—C62—C63	119.4 (5)
N15—C16—H16	107.8	C61—C62—H62	120.3
C19—C16—H16	107.8	C63—C62—H62	120.3
C17—C16—H16	107.8	C64—C63—C62	120.2 (6)
O18—C17—C16	105.5 (3)	C64—C63—H63	119.9
O18—C17—H171	110.6	C62—C63—H63	119.9
C16—C17—H171	110.6	C65—C64—C63	120.2 (6)
O18—C17—H172	110.6	C65—C64—H64	119.9
C16—C17—H172	110.6	C63—C64—H64	119.9
H171—C17—H172	108.8	C64—C65—C66	119.3 (6)
C16—C19—C22	111.1 (3)	C64—C65—H65	120.3
C16—C19—C20	108.4 (3)	C66—C65—H65	120.3
C22—C19—C20	110.3 (4)	C61—C66—C65	121.2 (5)
C16—C19—C21	107.7 (3)	C61—C66—H66	119.4
C22—C19—C21	110.3 (4)	C65—C66—H66	119.4
			
C6—N1—N2—C3	−1.4 (5)	C14—N15—C16—C19	−130.0 (4)
N1—N2—C3—N4	5.9 (6)	C14—N15—C16—C17	−2.9 (4)
N1—N2—C3—N7	−174.7 (3)	C14—O18—C17—C16	−3.5 (4)
C5—N4—C3—N2	−4.1 (5)	N15—C16—C17—O18	3.8 (4)
C5—N4—C3—N7	176.4 (3)	C19—C16—C17—O18	128.6 (4)
C8—N7—C3—N2	3.9 (6)	N15—C16—C19—C22	59.9 (5)
C8—N7—C3—N4	−176.7 (3)	C17—C16—C19—C22	−59.1 (5)
C3—N4—C5—C6	−1.9 (5)	N15—C16—C19—C20	−61.5 (4)
C3—N4—C5—C51	176.3 (3)	C17—C16—C19—C20	179.5 (3)
N2—N1—C6—C5	−4.2 (5)	N15—C16—C19—C21	−179.2 (3)
N2—N1—C6—C61	175.7 (3)	C17—C16—C19—C21	61.8 (5)
N4—C5—C6—N1	6.0 (5)	N4—C5—C51—C56	−148.3 (4)
C51—C5—C6—N1	−172.0 (3)	C6—C5—C51—C56	29.8 (6)
N4—C5—C6—C61	−174.0 (3)	N4—C5—C51—C52	26.6 (4)
C51—C5—C6—C61	8.0 (6)	C6—C5—C51—C52	−155.3 (3)
C3—N7—C8—C13	−12.4 (6)	C56—C51—C52—C53	0.0 (5)
C3—N7—C8—C9	168.3 (4)	C5—C51—C52—C53	−175.1 (3)
C13—C8—C9—C10	2.8 (5)	C51—C52—C53—C54	0.1 (6)
N7—C8—C9—C10	−177.8 (3)	C52—C53—C54—C55	−0.4 (7)
C13—C8—C9—C14	−178.7 (3)	C53—C54—C55—C56	0.5 (7)
N7—C8—C9—C14	0.7 (5)	C54—C55—C56—C51	−0.4 (6)
C8—C9—C10—C11	−1.1 (6)	C52—C51—C56—C55	0.1 (5)
C14—C9—C10—C11	−179.6 (4)	C5—C51—C56—C55	174.9 (3)
C9—C10—C11—C12	−0.7 (6)	N1—C6—C61—C66	54.0 (5)
C10—C11—C12—C13	0.8 (7)	C5—C6—C61—C66	−126.1 (4)
C11—C12—C13—C8	0.9 (6)	N1—C6—C61—C62	−125.2 (4)
N7—C8—C13—C12	177.9 (4)	C5—C6—C61—C62	54.7 (5)
C9—C8—C13—C12	−2.8 (6)	C66—C61—C62—C63	1.4 (6)
C16—N15—C14—O18	0.8 (5)	C6—C61—C62—C63	−179.4 (4)
C16—N15—C14—C9	−178.1 (3)	C61—C62—C63—C64	0.2 (8)
C17—O18—C14—N15	1.9 (4)	C62—C63—C64—C65	−0.7 (9)
C17—O18—C14—C9	−179.1 (3)	C63—C64—C65—C66	−0.4 (8)
C10—C9—C14—N15	179.6 (4)	C62—C61—C66—C65	−2.5 (6)
C8—C9—C14—N15	1.2 (6)	C6—C61—C66—C65	178.3 (4)
C10—C9—C14—O18	0.7 (5)	C64—C65—C66—C61	2.0 (7)
C8—C9—C14—O18	−177.7 (3)		

### Hydrogen-bond geometry (Å, °)

**Table:**

*D*—H···*A*	*D*—H	H···*A*	*D*···*A*	*D*—H···*A*
N7—H7···N15	0.97 (4)	1.87 (5)	2.671 (4)	138 (4)
C13—H13···N2	0.93	2.31	2.919 (5)	122
C53—H53···O18^i^	0.93	2.54	3.250 (5)	133

Symmetry codes: (i) −*x*+1, *y*−1/2, −*z*+3/2.

## References

[bb1] Bruker (2005). *APEX2*, *SAINT* and *SADABS* Bruker AXS Inc., Madison, Wisconsin, USA.

[bb2] Castro, J., Cabaleiro, S., Perez-Lourido, P., Romero, J., Garcia-Vazquez, J. A. & Sousa, A. (2001). *Polyhedron*, **20**, 2329–2337.

[bb3] Coeffard, V., Müller-Bunz, H. & Guiry, P. J. (2009). *Org. Biomol. Chem.* **7**, 1723–1734. 10.1039/b822580j 19343263

[bb4] Desimoni, G., Faita, G. & Jørgensen, K. A. (2006). *Chem. Rev.* **106**, 3561–3651. 10.1021/cr0505324 16967916

[bb5] Farrugia, L. J. (1997). *J. Appl. Cryst.* **30**, 565.

[bb6] Farrugia, L. J. (1999). *J. Appl. Cryst.* **32**, 837–838.

[bb7] Hargaden, G. C. & Guiry, P. J. (2009). *Chem. Rev* **109**, 2505–2550. 10.1021/cr800400z 19378971

[bb8] Lindsey, C. W. & Layton, M. E. (2004). *Science of Synthesis*, *Houben–Weyl Methods of Molecular Transformation*, Vol. 17, p. 357. Stuttgart: *Georg Thieme Verlag.*

[bb9] Sheldrick, G. M. (2008). *Acta Cryst.* A**64**, 112–122. 10.1107/S0108767307043930 18156677

